# Association between IGF‐1 levels ranges and all‐cause mortality: A meta‐analysis

**DOI:** 10.1111/acel.13540

**Published:** 2022-01-20

**Authors:** Jamal Rahmani, Alberto Montesanto, Edward Giovannucci, Hamid Zand, Meisam Barati, John J. Kopchick, Mario G. Mirisola, Vincenzo Lagani, Hiba Bawadi, Raffaele Vardavas, Alessandro Laviano, Kaare Christensen, Giuseppe Passarino, Valter D. Longo

**Affiliations:** ^1^ Department of Community Nutrition Faculty of Nutrition and Food Technology National Nutrition and Food Technology Research Institute Shahid Beheshti University of Medical Sciences Tehran Iran; ^2^ Department of Biology, Ecology and Earth Sciences University of Calabria Rende Italy; ^3^ Departments of Nutrition Harvard TH Chan School of Public Health Boston Massachusetts USA; ^4^ National Nutrition and Food Technology Research Institute Faculty of Nutrition Sciences and Food Technology Shahid Beheshti University of Medical Sciences Tehran Iran; ^5^ Department of Biomedical Sciences Heritage College of Osteopathic Medicine Ohio and Edison Biotechnology Institute Ohio University Athens Ohio USA; ^6^ Department of Surgical, Oncological and Stomatological Disciplines Università di Palermo Palermo Italy; ^7^ Institute of Chemical Biology Ilia State University Tbilisi Georgia USA; ^8^ Biological and Environmental Sciences and Engineering Division (BESE) King Abdullah University of Science and Technology KAUST Thuwal Saudi Arabia; ^9^ Human Nutrition Department College of Health Sciences QU‐Health Qatar University Doha Qatar; ^10^ RAND Corporation Santa Monica California USA; ^11^ Department of Translational and Precision Medicine Sapienza University Rome Italy; ^12^ Danish Aging Research Center University of Southern Denmark Odense Denmark; ^13^ Longevity Institute Davis School of Gerontology and Department of Biological Sciences University of Southern California Los Angeles California USA; ^14^ IFOM FIRC Institute of Molecular Oncology Milan Italy

**Keywords:** IGF‐1, mortality, protein intake

## Abstract

The association between IGF‐1 levels and mortality in humans is complex with low levels being associated with both low and high mortality. The present meta‐analysis investigates this complex relationship between IGF‐1 and all‐cause mortality in prospective cohort studies. A systematic literature search was conducted in PubMed/MEDLINE, Scopus, and Cochrane Library up to September 2019. Published studies were eligible for the meta‐analysis if they had a prospective cohort design, a hazard ratio (HR) and 95% confidence interval (CI) for two or more categories of IGF‐1 and were conducted among adults. A random‐effects model with a restricted maximum likelihood heterogeneity variance estimator was used to find combined HRs for all‐cause mortality. Nineteen studies involving 30,876 participants were included. Meta‐analysis of the 19 eligible studies showed that with respect to the low IGF‐1 category, higher IGF‐1 was not associated with increased risk of all‐cause mortality (HR = 0.84, 95% CI = 0.68–1.05). Dose–response analysis revealed a U‐shaped relation between IGF‐1 and mortality HR. Pooled results comparing low vs. middle IGF‐1 showed a significant increase of all‐cause mortality (HR = 1.33, 95% CI = 1.14–1.57), as well as comparing high vs. middle IGF‐1 categories (HR = 1.23, 95% CI = 1.06–1.44). Finally, we provide data on the association between IGF‐1 levels and the intake of proteins, carbohydrates, certain vitamins/minerals, and specific foods. Both high and low levels of IGF‐1 increase mortality risk, with a specific 120–160 ng/ml range being associated with the lowest mortality. These findings can explain the apparent controversy related to the association between IGF‐1 levels and mortality.

## Graphical abstract

The analysis of different ranges of IGF‐1, in more than 30,000 subjects, suggests that both high and low levels of IGF‐1 increase mortality risk, with a specific mid range being associated with the lowest mortality. Using the NHANES III survey, we show an association between high intake of animal proteins, carbohydrate, and milk‐based products and IGF‐1 levels. These data, together with a number of randomized clinical trials, can point to nutritional strategies to modulate IGF‐1 levels.

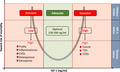

## 1 INTRODUCTION

Insulin‐like growth factors (IGFs) are proteins with multiple functions including stimulation of cell proliferation, inhibition of apoptosis, and enhancement of cell motility as well as the regulation of cell differentiation and transformation (Delafontaine et al., 2004). Among IGFs, circulating IGF‐1, which is mainly synthesized in the liver in response to stimulation by growth hormone (GH) through its receptor (GHR), mediates many of the pro‐growth effects of GH. In the bloodstream, the majority of the IGF‐1 is found in a binary complex with IGFBP (mainly IGFBP‐3) proteins or a ternary complex including also the glycoprotein Acid Labile Subunit (Juul et al., 1995). IGF‐1R belongs to the tyrosine kinase receptor family and triggers a signal transduction cascade involving PI3K, AKT, and TOR (Delafontaine et al., 2004).

A series of studies have shown that high levels of IGF‐1 are associated with an increased risk of tumors including prostate, pre‐ and postmenopausal breast, lung, thyroid, and colorectal cancers (Ma et al., 1999; Renehan et al., 2004; Shi et al., 2001). An increase in serum IGF‐1 level of 100 ng/ml was shown to correspond to a 69% increase in colorectal cancer risk (Ma et al., 1999). High levels of IGF‐1 were also shown to be associated with a 49% increase in prostate cancer, 65% increase in breast cancer (Renehan et al., 2004), and a 106% increase in lung cancer risk (Yu et al., 1999). Furthermore, in worms, flies, and mice insulin/IGF‐1 signaling reduces lifespan and healthspan (Bartke et al., 2013; Fontana et al., 2010; Kenyon, 2010; Podshivalova et al., 2017). On the contrary, several studies have found a connection between low levels of IGF‐1 and conditions such as cardiovascular diseases (CVD), diabetes mellitus, osteoporosis, and sarcopenia (Garnero et al., 2000; Higashi et al., 2010).

Among studies focused on the relationship between IGF‐1 levels and mortality, some reported no relationship (Brugts et al., 2008; Hu et al., 2009), whereas others showed a positive association between high levels of IGF‐1 and mortality (Andreassen et al., 2009; Colombo et al., 2017; Duggan et al., 2013) and four indicated that low levels of IGF‐1 are associated with higher mortality (Cappola et al., 2003; Friedrich et al., 2009; Jia et al., 2014; Miyake et al., 2016). Interestingly, a meta‐analysis carried out by Burgers et al. in 2011 had suggested a U‐shaped relation between circulating IGF‐1 and mortality (Burgers et al., 2011).

Because serum IGF‐1 level measurements are common in the clinic, to address these controversial findings and identify an IGF‐1 range consistently associated with lower mortality, we conducted a dose–response meta‐analysis of prospective cohort studies assessing the relationship between IGF‐1 levels and all‐cause mortality. The third national health and nutrition examination survey (NHANES III, 1988–1994) data were instead used to identify nutrients whose intake affects IGF‐1 levels.

## 2 RESULTS

### 2.1 Study selection

**FIGURE 1:**
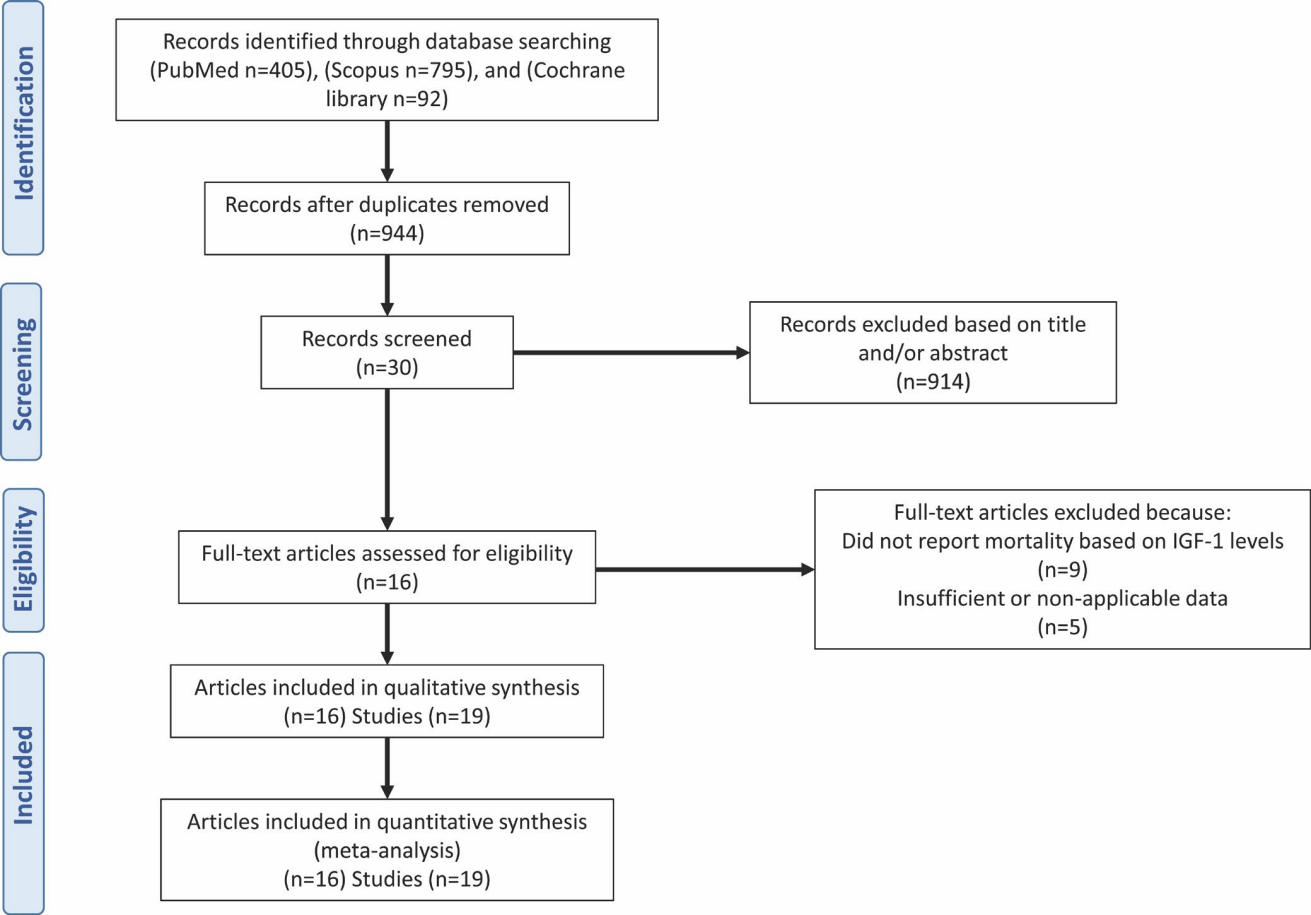
Literature search and study selection process for the systematic review and meta‐analysis

Figure 1 shows the full process of literature search and study selection. A total of 1292 reports were initially identified. After removing duplicates, 943 articles remained. 914 records were excluded based on the title and/or abstract. Fourteen studies were excluded after full‐text review: Nine studies did not report HR for mortality in the IGF**‐**1 categories, and five studies did not categorize IGF‐1 levels. As a consequence, 19 independent studies in 16 articles were included in this meta‐analysis (see Table S2).

### 2.2 Study characteristics and quality assessment

Baseline characteristics of the 19 eligible studies with 30,876 participants are presented in Table 1. These studies were published between 2007 and 2019. Mean age of participants at baseline was 65 years with a mean follow‐up duration of about 7 years, ranging from 2.3 to 12 years. Studies were conducted in Germany, Netherlands, the United States, UK, Sweden, Japan, Australia, Denmark, China, and Italy. According to the Newcastle–Ottawa Quality Assessment scale, 12 studies had superior quality (Supporting information p8).

**TABLE 1:** Baseline characteristics in the meta‐analysis on the association between IGF‐1 levels and risk of all‐cause mortality (30,876 participants)

Author	Year	Country population	Cohort name Follow‐up (year)	Sex (1: women, 2: men, 3: both)	Death (*n*)	Person/years	Population	Age (years) range
Friedrich, N	2009	Germany	MONICA (8.5)	2	240	15,861	1988	51 –
				1	108	17,491	2069	48 ‐
Van Bunderen, C	2010	Netherlands	LASA (11.6)	3	633	–	1273	74 55–85
Friedrich, N	2011	Germany	DETECT (6)	2	131	7737	2463	59 –
				1	102	11,392	3603	57 18–95
Duggan, C	2013	USA	HEAL (8)	1	87	–	600	57 40–64
Rowlands, M	2012	UK	Royal Hallamshire Hospital (3.7)	2	27	741	396	70 –
Kaplan, R	2017	USA	CHS (8)	3	722	13,930	2268	78 68–102
Sun, J	2016	Sweden	Karolinska University (2.3)	3	149	–	543	53 19–87
Miyake, H	2016	Japan	Shimane University (6.6)	1	25	–	382	67 –
				2	46	–	468	64 –
Svensson, J	2012	Sweden	MrOS (6)	2	111	–	2101	75 69–81
Yeap, B	2011	Australia	HIMS (5.2)	2	694	–	3983	77 70–89
Andreassen, M	2009	Denmark	– (5)	3	103	–	642	68 50–89
Brugts, M	2008	Netherlands	Zoetermeer (8.6)	3	170	–	376	71 73–94
Arai, Y	2008	Japan	Tokyo Centenarians (6)	3	–	611	252	101.5 100–108
Saydah, Sh	2007	USA	NHANES III (12)	3	743	–	6056	43 –
Shen, L.	2018	China	– (3)	3	–	–	216	54 46–63
Maggio, M	2013	Italy	CHIANTI (8)	3	240	–	1197	69 65<

### 2.3 Main results of meta‐analysis

**FIGURE 2:**
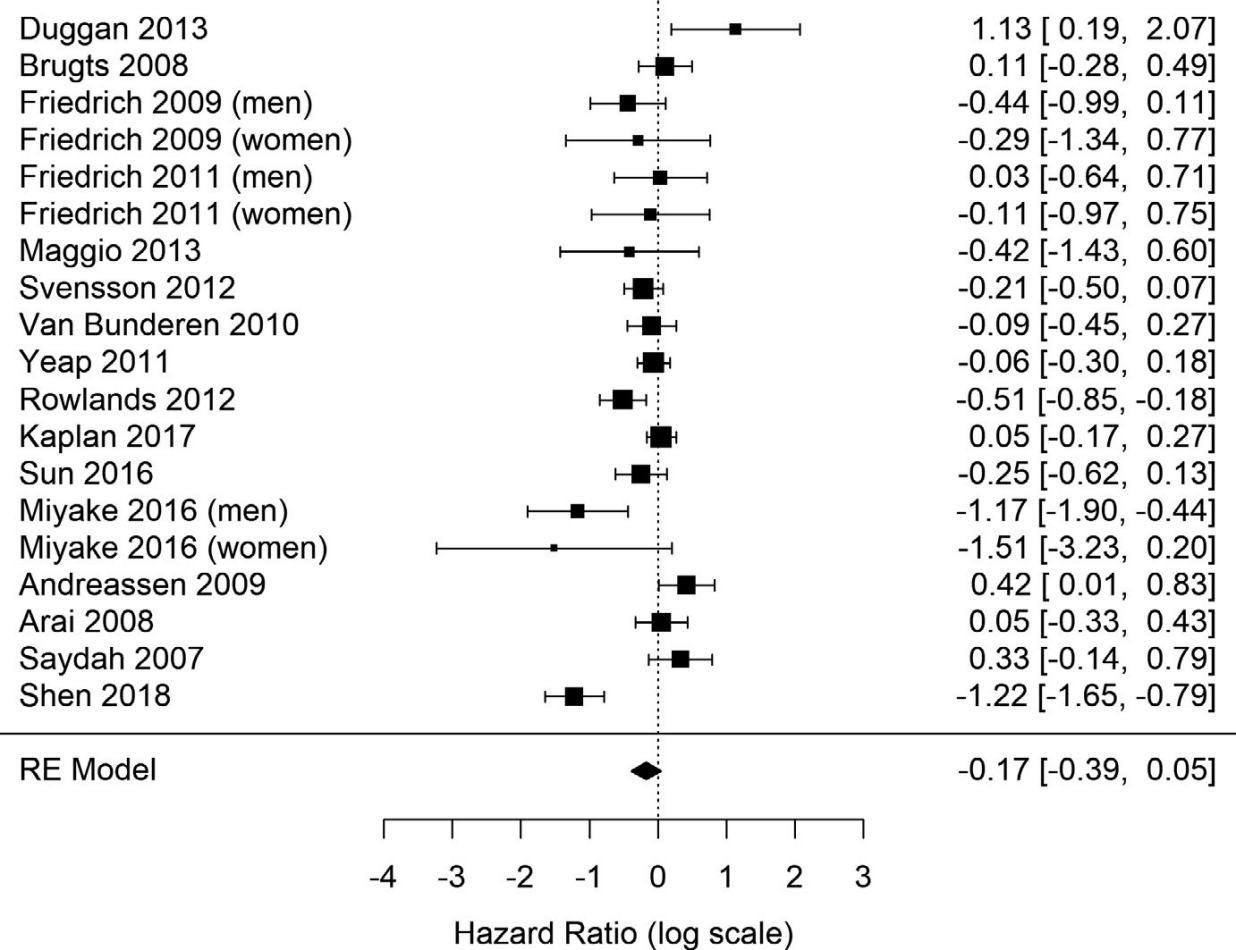
Forest plots showing the meta‐analytic estimate for IGF‐1 levels and mortality (highest vs. lowest IGF‐1 categories)

Meta‐analysis of the 19 eligible studies showed that with respect to the low IGF‐1 category, higher IGF‐1 was not associated with increased risk of all‐cause mortality (HR = 0.84, 95% CI = 0.68–1.05) (Figure 2), with a high heterogeneity detected among studies (*Q* = 65.4, *p*‐value < 0.0001; *I* ^2^ = 78.31%).

### 2.4 Dose–response meta‐analysis

**FIGURE 3:**
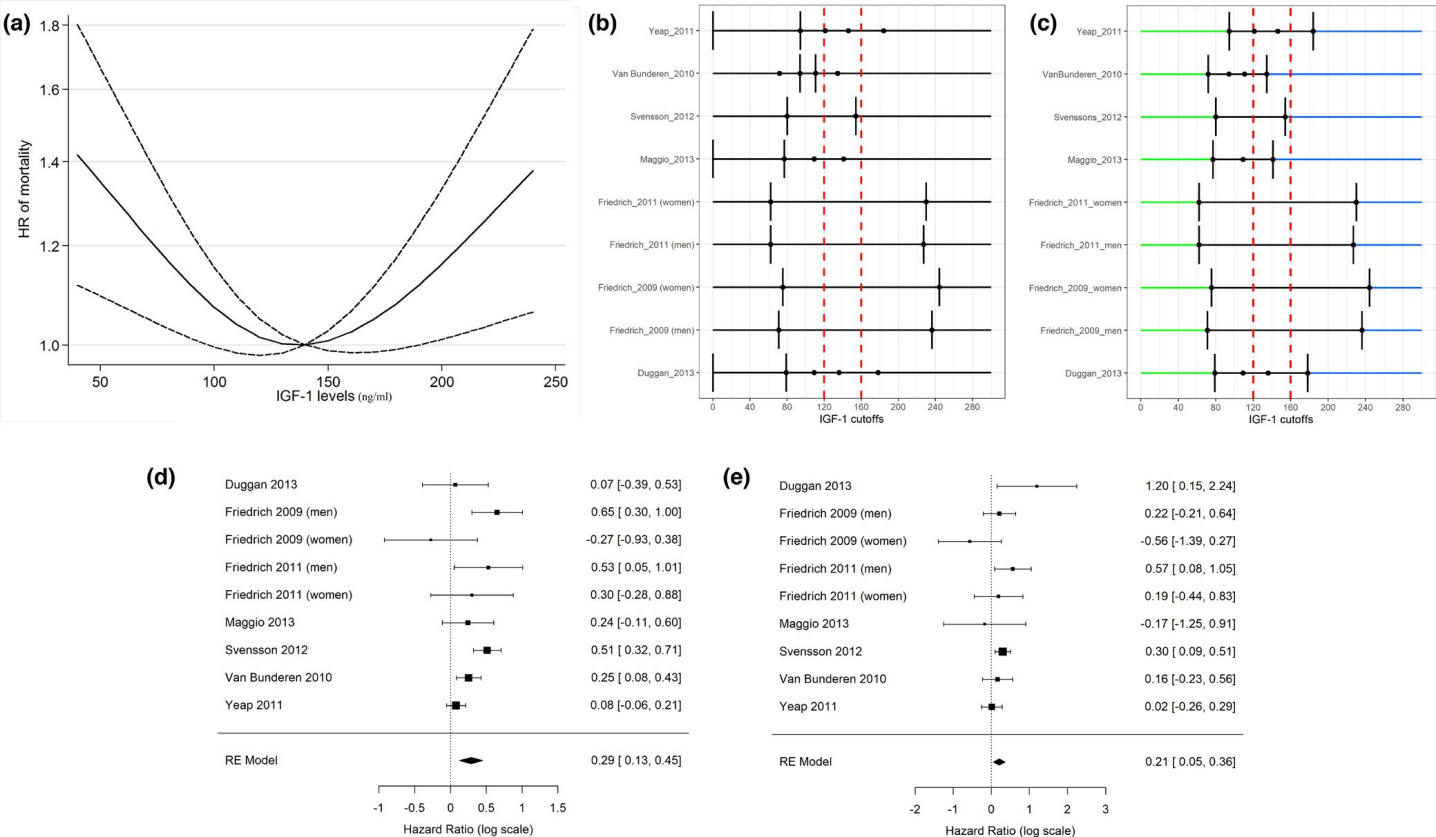
(a) Dose–response association between IGF‐1 levels and risk of all‐cause mortality (P_1_ for nonlinearity = −0.0056, Coef_1_ = 0.001; P_2_ for nonlinearity < 0.001, Coef_2_ = 0.0105). Dashed lines indicate the 95% confidence intervals for the spline model. The horizontal dashed line corresponds to the reference (140 ng/ml) HR of 1.0. (b, c): IGF‐1 categories originally used to define high‐, low‐, and intermediate‐risk intervals in the nine selected studies (b). Vertical bars correspond to the adopted IGF‐1 cutoffs. (c) Reconfiguration of the IGF‐1 categories according to the proposed approach. Blue lines define high risk categories; green lines define low risk categories. The combined HR was obtained using the intermediate categories as a reference group (black lines). *For both Friedrich’ studies IGF‐1 cutoffs were computed as mean values of the groups identified according to the age‐ and sex‐specific 10th and 90th percentile. (d, e) Forest plot of pooled analysis for the low (d) and the high (e) categories of IGF‐1 compared to the middle category (hazard ratios reported in the log scale in both panels)

Nine studies in seven papers with 18,492 participants and 3422 deaths reported sufficient detailed information for nonlinear dose–response meta‐analyses of IGF‐1 levels and all‐cause mortality. Mean follow‐up duration was 7.5 years ranging from 5.2 to 11.6 years. Dose–response analysis revealed a U‐shaped relation between IGF‐1 and HR of mortality with both high and low values of IGF‐1 associated with an increased the risk of mortality (Figure 3a).

Because several among the 19 eligible studies indicate that both low and high IGF‐1 levels are associated with increased mortality, and based on the dose–response analysis (Figure 3a), we selected nine studies that used at least an intermediate category of IGF‐1 that overlapped with the range of IGF‐1 levels that the dose–response analysis indicated to be associated with the lowest mortality (120–160 ng/ml, Figure 3b,c). These studies were used to compare low/high vs. middle categories of IGF‐1 with respect to the risk of all‐cause mortality. Meta‐analysis showed that lower IGF‐1 levels were associated with increased risk of mortality compared to mid‐range IGF‐1 (HR = 1.33, 95% CI = 1.14–1.57; Figure 3d), but with a significant heterogeneity observed among the analyzed studies (*I* ^2^ = 62.85%; *Q* = 23.0, *p*‐value = 0.003). The same nine studies showed that higher IGF‐1 levels, which are not associated with increased mortality when compared to low IGF‐1 levels, but were associated with increased risk of all‐cause mortality compared to the mid‐range IGF‐1 (HR = 1.23, 95% CI = 1.06–1.44; Figure 3e). There was no obvious heterogeneity among the nine studies reporting HRs comparing high vs. middle categories of IGF‐1 levels (*I* ^2^ = 4.45%; *Q* = 12.08, *p*‐value = 0.148). Similar results were obtained for studies including participants older than 70 years (HR = 1.18, 95% CI 0.98–1.43 for high vs. medium IGF‐1 categories; HR = 1.31, 95% CI = 1.07–1.59, for low vs. medium IGF‐1 categories), again with an elevate heterogeneity among studies (*Q* = 13.37, *p*‐value = 0.004; *I* ^2^ = 75.54%). However, it is worth noticing that in subjects older than 70 years the association between IGF‐1 and increased mortality appears to be weaker with borderline statistical significance. Adopting a larger range of IGF‐1 (100–180 ng/ml) does not change the main findings of the analysis (supporting information pp 2–3).

### 2.5 Publication bias

Funnel plot of all‐cause mortality is shown in Supporting information (p 3). No publication bias was found among studies on all‐cause mortality (*p*‐values = 0.41 and 0.49 for Egger's and Begg's tests, respectively). Sensitivity analysis did not show significant differences beyond the limits of 95% CI among studies (Supporting information p4).

### 2.6 Epidemiological diet study

Because nutrition is perhaps the most relevant modulator of IGF‐1 levels (Key, 2011; Levine et al., 2014; Watling et al., 2021), third national health and nutrition examination survey (NHANES III, 1988–1994) data were used to investigate the relationship between IGF‐1 and the daily intake of specific nutrients. The study population included 1152 men and 1453 women. Mean age of participants was 45.11 ± 17.97 years.

Mean serum IGF‐1 concentrations increased in subjects reporting a higher protein or carbohydrate intake (*p*‐value = 0.007). The intake of vitamins and minerals was also associated with higher IGF‐1 levels (Table 2A,B). We next determined the type of food whose consumption was correlated to circulating serum IGF‐1. High consumption of dairy products including milk, cheese and yogurt, and margarines was associated with increased IGF‐1 levels in agreement with previous studies, while high consumption of butter, eggs, and egg products was associated with decreased levels of IGF‐1 (Giovannucci et al., 2003) (Table 2C).

**TABLE 2:** Associations between quintiles of macronutrient (a), micronutrient (b) and food (c) intakes and IGF‐1 serum concentrations (2605 participants)

Nutrients	IGF−1 (ng/ml)		*p*‐value
	First quintile	Fifth quintile	
A: Macronutrients			
Protein	232.8	241.3	0.007
Animal protein	230.3	244.2	0.001
Plant protein	227.9	238.0	0.110
Carbohydrate	226.3	238.7	0.007
Total fat	234.0	237.6	0.171
Saturated fat	232.9	238.4	0.115
Polyunsaturated fat	232.4	243.4	0.062
Monounsaturated fat	234.2	236.3	0.330
B: Micronutrients			
Retinol	229.4	244.3	<0.001
Beta‐carotene	230.6	245.9	0.001
Vitamin B1 (thiamine)	223.5	241.3	0.002
Vitamin B2 (riboflavin)	226.8	245.8	<0.001
Vitamin B6	229.5	240.4	0.012
Vitamin B12	226.8	247.1	<0.001
Vitamin C	227.0	240.6	0.001
Vitamin D	224.5	248.6	<0.001
Calcium	230.5	246.3	0.001
Iron	227.8	244.2	0.005
Magnesium	226.0	240.3	0.009
Phosphorus	231.2	240.6	0.023
Potassium	227.4	244.2	<0.001
C: Foods			
Eggs and egg products	236.7	224.1	0.010
Milk and milk beverages	233.9	249.0	0.006
Cheese and fromage blanc	232.0	246.7	<0.001
Yogurt	233.0	245.7	0.002
Meat	234.0	238.8	0.256
Processed meat	235.8	232.5	0.454
Poultry	234.9	237.4	0.563
Fish and shellfish	234.3	239.7	0.183
Vegetables	234.7	236.3	0.692
Fruits	233.3	241.7	0.041
Potatoes and other tubers	235.2	235.8	0.902
Cereal and cereal products	234.3	237.8	0.401
Butter	237.2	226.9	0.014
Margarines	234.5	249.5	0.033

## 3 DISCUSSION

The role of IGF‐1 on mortality is controversial with studies showing either increased or decreased mortality in the high IGF‐1 compared to low IGF‐1 group. This meta‐analysis of 19 cohort studies with 30,876 participants confirms the absence of a significant difference in the HRs of all‐cause mortality with IGF‐1 levels when the highest and lowest IGF‐1 levels are compared. This nonlinear dose–response meta‐analysis supports a U‐shaped relationship for serum IGF‐1 concentration and mortality with the lowest mortality associated with the 120–160 ng/ml range. Since there was an obvious difference in the IGF‐1 cutoff levels used to define high‐, low‐, and intermediate‐risk categories, we attempted to rule out this source of heterogeneity by standardizing serum IGF‐1 categories. This approach revealed that both the lowest and highest categories of IGF‐1 had a significantly higher risk of all‐cause mortality compared with middle category. Furthermore, a direct relation was found between foods associated with growth including proteins, carbohydrates, milk‐based products, and B vitamins and IGF‐1 levels, in agreement with clinical studies indicating that optimal range levels of this growth factor can be achieved by dietary changes.

In mice, very low levels of IGF‐1 are associated with reduction in a range of diseases and conditions including cancer, diabetes, and cognitive decline and with record longevity (Bartke et al., 2013). In fact, mice with severe IGF‐1 deficiency, achieved by either growth hormone receptor (GHRD) or GH deficiency, display a 40% extended longevity (Bartke et al., 2013). Also, GHRD mice are protected from age‐related decline in memory and perform similarly to young normal mice. Additionally, insulin/IGF‐signaling (IIS) pathway accelerates aging in *Caenorhabditis elegans* and the fly *D*. *melanogaster* (Bartke et al., 2013).

In humans, studies on patients with Laron syndrome (LS), whose IGF‐1 levels are extremely low, have reported a reduction in pro‐aging signaling, cancer, diabetes, and cognitive decline (Guevara‐Aguirre et al. 2011; Nashiro et al. 2017). Steuerman et al. (2011) also surveyed 230 individuals with LS and found no cases of cancer.

On the contrary, other studies reported an association between low levels of IGF‐1 and conditions like CVD, diabetes mellitus, osteoporosis, and sarcopenia although a causal relationship has not been established (Brioche et al., 2014; Katsanos et al., 2001; Lenk et al., 2010; Saki et al., 2017).

Several studies have also demonstrated that IGF‐1 has a protective effect against CVD. However, local expression of IGF‐1 rather than circulating IGF‐1 could be responsible for this protective effect (Vinciguerra et al., 2009, 2012).

These apparently, or potentially, opposite effects of IGF‐1 generated a controversy related to the value of measuring IGF‐1 levels to assess disease and mortality risk in patients. On the basis of the available data, we propose that although low IGF1 may be healthy if it is maintained in the ideal 120–160 ng/ml range by dietary regulation = the reduction of IGF‐1 levels is due to frailty and sickness together with inflammatory diseases could explain the increased mortality in patients with low or very low IGF‐1 levels. Notably, bioavailability and local levels of IGF‐1 are affected by inflammation. For example, GH resistance of liver cells caused by chronic inflammation can lead to decreased expression of IGF‐1 (DeBoer et al., 2017; Street et al., 2004, Street et al. (2006). Malnutrition also directly inhibits GH binding to GH receptors and is associated with GH resistance (Yamamoto et al., 2013).

Generally, IGF‐1 levels decrease from age 20 to the end of life. The diet and particularly proteins, dietary restrictions, obesity, and lifestyle influence the level of IGF‐1. In fact, a study investigating the relationship between protein consumption and mortality discovered a significant and direct relationship between high protein intake, high IGF‐1 levels, and increased hazard ratios for all‐cause mortality (Willett & Ludwig, 2020). However, this association was no longer observed in the over 65 population, in agreement with the much lower IGF‐1 levels in the over 65 subjects independently of their protein intake (Levine et al., 2014) and in line with other reports (Willett & Ludwig, 2020). We also report that the intake of milk and other nutrients important for growth including carbohydrates and B vitamins are associated with higher IGF‐1 levels.

In summary, because extensive data in both mice and humans consistently show that even extremely low levels of IGF‐1 are associated with increased lifespan or healthspan and in agreement with the meta‐analysis presented here, we propose that low‐ to mid‐range levels of IGF‐1 (120–160 ng/ml) are reflective of the “healthiest” status. Burgers et al. (2011) have previously proposed that the relation between circulating IGF‐1 and mortality is U‐shaped, based on a meta‐analysis of studies that are all over 10 years old and that represent 14,906 individuals. Such proposal, unfortunately, has not been supported by subsequent studies. Here, thanks to many additional studies which have been carried out on this topic since the Burgers paper, we report a much larger data set (30,876 individuals) which allowed more robust conclusions. In addition, thanks to the statistical approach we used and also to studies we and others carried out in mice and humans that with extremely low IGF‐1 levels (GHDs, GHRDs) we, for the first time, have provided a reasonable explanation for the U‐shape relationship between IGF‐1 and mortality, which also allowed us to propose an ideal IGF‐1 range, which could be used by physicians as a reference. Finally, unlike previous reviews and meta‐analyses, we provide a link between the type of food consumed by the US population and IGF‐1 levels based on CDC NHANES data. In fact, based on NHANES database results, a total protein intake in the 50–80 g/day in the US population is associated with the ideal IGF‐1 range described here. This compares to the approximately 91 ± 22 g/day average protein intake in the US population aged 19–30 years, and to the approximately 66 ± 17 g/day average protein intake in the elderly US population (Fulgoni, 2008), indicating that the majority of adults living in the US consume excessive levels of proteins, whereas the elderly may be consuming an appropriate level.

Two strengths of the present meta‐analysis study are (i) incorporation of prospective cohort studies and (ii) standardization of serum IGF‐1 categories in studies with different cut‐points to better extrapolate the results (iii) utilization of high‐quality studies that had a large sample and a long duration of follow‐up. Limitations of the present study are as follows: (i) limited access to fundamental information of some articles that as a consequence was not included in the dose–response section analysis; (ii) the association between IGF‐1 levels and all‐cause mortality may be due to other potential confounding factors not taken into account; (iii) the choice of the IGF‐1 interval for standardization of IGF‐1 categories could be different. However, considering that previous studies reported a U‐shaped relationship between IGF‐1 and mortality, even with a different interval window (for instance larger) the results of this approach would not change; (iv) it is not clear which range of IGF‐1 below the 120 ng/ml is associated with the increased mortality. In fact, these data do not allow us to determine whether the populations with IGF‐1 levels below 120 ng/ml may be heterogeneous and contain both individuals with high and low mortality risk. Not surprisingly, the heterogeneity test in the low‐ vs. mid‐range categories of IGF‐1 suggests high heterogeneity among the populations included. This was not observed in the mid vs. high comparison.

An additional limitation of the study may be that in seven studies sick subjects were included, due to cancer, diabetes, and CKD. However, sick subjects accounted for 15.2% of the total sample (4706 out of 30,876, see Table 1) and that they had a minor influence on the final results, as it emerges from the Forest plot (see Figure 2).

In support of the role of the association between low growth factors and longevity, animal studies indicate that the longevity advantage of smaller mammals can be considerable. The longevity advantage of smaller mammals is strongly supported by studies of domestic dogs with large differences in adult body size between dog breeds with smaller size being associated with shorter period of growth, fewer pups per litter, and longer life (Jimenez, 2016).

An important aspect to take into account is the time at which IGF‐1 is measured. In particular, in mice it has been observed that GH treatment limited to a few weeks during development show a significant impact on longevity and related outcome (Sun et al., 2017) and that late‐life targeting of the IGF‐1 receptor improves healthspan and lifespan especially in females (Mao et al., 2018). While we recognize this very important limitation, we would like also to highlight that from an analytic point of view the information about the time at which IGF‐1 is measured is not a specific problem of our meta‐analysis study, but in general of the studies on which our meta‐analysis is based. It is worth mentioning that the age range of the subjects included in the analysis was 18–108 years and that eight studies, accounting for 17,538 subjects (56.8% of the total), had a median age below 60 years. 96% of the subjects had a follow‐up period of 5 years or more, and more than half of them (52%) had a follow‐up of 8 years or more. In addition, similar results were obtained in the whole sample and in the subjects older than 70 years of age.

In conclusion, by analyzing and comparing different ranges of IGF‐1 in 30,876 subjects, we find that both high and low levels of IGF‐1 increase mortality risk, and for the first time, we identify a specific mid‐range being associated with the lowest mortality (120–160 ng/ml). Using the NHANES III survey, we show an association between high intake of animal proteins, carbohydrates, and milk‐based products and IGF‐1 levels. These results can point to diagnostic, nutritional, and pharmacological strategies to optimize IGF‐1 levels and help reduce mortality.

## 4 EXPERIMENTAL PROCEDURE

### 4.1 Search strategy

A systematic literature search was conducted by two independent reviewers (JR and MB) in PubMed/MEDLINE, Scopus, and Cochrane Library up to September 2019, using a combination of Mesh terms and keywords (Supporting information p5), and discrepancies were resolved and confirmed using a third investigator (HZ).

In order to find more relevant studies, a manual search was done among the reference lists of retrieved articles. The search was limited to cohort studies in English language that were carried out in humans. An email alert service was created and enabled to identify new articles published after our search. We followed the Meta‐analysis of Observational Studies in Epidemiology (MOOSE) study guidelines for conducting dose–response meta‐analyses and reporting results (Supporting information pp 9–10).

### 4.2 Selection criteria

The PICOS criteria were used to establish study eligibility. These PICOS criteria were as follows: patients: general population of adult; intervention (intervention, prognostic factor, or exposure): IGFs levels; control (control or comparison): risk ratios (RR), hazard ratios (HR), or odds ratios (OR) of mortality; outcome: mortality; study design: All types of observational studies. Two authors (JR and MB) independently reviewed the abstract of all articles in order to select eligible studies. The two investigators independently reviewed the full‐text of relevant articles. Published studies were eligible for inclusion in this meta‐analysis if they were written in English, had a prospective cohort design, reported a hazard ratio with 95% CI for two or more categories of IGF‐1 and were conducted among adults (age ≥18 years).

### 4.3 Data extraction

Two reviewers (JR and MB) independently extracted the data using a predefined data extraction form, and discrepancies were discussed and eventually resolved and confirmed by the third reviewer (HZ). We extracted the following data from each article: first author's name, publication year, study location (country), duration of follow‐up in years, sex, age, total population, number of deaths, IGF‐1 categories, covariates adjusted for in the multivariable analysis, and hazard ratios (HR) with their 95% CIs for all categories of circulating IGF‐1. To this purpose, the greatest degree of adjustment model for potentially confounding variables was taken into account (Supporting information pp. 6–7).

For each study, the mean circulating IGF‐1 for each quantile was coupled with the corresponding HR. If the mean IGF‐1 per quantile was not reported in the article, the average of upper and lower boundaries of each quantile was considered as the mean of that quantile. Likewise, if the total number of people and the number of deaths per each quantile were not reported in the article, an email was sent to the corresponding author to request the missing information. The quality of each article was assessed by Newcastle–Ottawa Quality Assessment scale (Stang, 2010).

### 4.4 Epidemiological diet study

Third national health and nutrition examination survey (NHANES III, 1988–1994) data were used to investigate the relation between IGF‐1 blood concentration and nutrient intake (Madsen et al., 2019; United States Department of Health and Human Services. Centers for Disease Control and Prevention. National Center for Health Statistics, 1998). Nutrient intake and IGF‐1 levels were determined by a 24‐h dietary recall and ELISA, respectively. We used adult data set whose participants were 30 years old or more. Participants with missing data in IGF‐1 levels, age, gender, and participants with cancer or diabetes were excluded from data set. Furthermore, we excluded participants whose total daily energy intake was outside the credible range (men <800 or >4200 kcal/day, women <600 or >3500 kcal/day) (Feskanich, 2000).

### 4.5 Statistical analyses

Firstly, we used a random‐effects model with a restricted maximum likelihood heterogeneity variance estimator to determine the HR for the highest vs. lowest category of circulating IGF‐1. The heterogeneity among studies was estimated by the Cochran *Q* test and *I* ^2^ statistic. Heterogeneity was confirmed with a significance level <.10. Publication bias was assessed visually with funnel plots and using Egger’s regression and Begg’s rank correlation tests. Sensitivity analysis was performed to investigate the effect of each study on overall analysis.

Secondly, dose–response analysis (potential of nonlinear association) was examined by modeling circulating IGF‐1 levels using restricted cubic splines with three knots at fixed percentiles (10, 50, and 90%) of the distribution. A *p*‐value for nonlinearity of the dose–response meta‐analysis was calculated based on the null hypothesis that the coefficient of the second spline was equal to zero. Thirdly, since categories defined on percentiles might make difficult to compare associations or effects across studies, we defined an optimal interval of serum IGF‐1 based on dose–response meta‐analysis. All studies with at least one IGF‐1intermediate category overlapping the IGF‐1 optimal interval were included in the subgroup analysis. Conversely, studies availing of open‐ended categories encompassing a broad range of exposure or confounder effects were excluded. For studies with more than one middle‐risk group, we combined the corresponding risk estimates with inverse variance weights, obtaining exactly three risk categories (high, middle, and low) for each study. For studies in which the reference category was not the middle one, we used the approach described in Orsini (2010) for recalculating risk estimates assuming the middle category as reference (Orsini, 2010). Then, we combined the HR across the selected studies by using a random‐effects model with a heterogeneity variance estimator based on restricted maximum likelihood.

Finally, least‐square means of serum IGF‐1 across quintiles of dietary variables were estimated by using linear regression models. A test for linear trend was performed to assess the effect of dietary categories by scoring the categories according to their median value and entering the variable as a continuous term in the ANOVA. Due to their effect on IGF‐1 levels, age and gender were used as covariates. In addition, also total energy intake was included in these models to partially control for error in estimated diet intake. A *p*‐value of <0.05 was considered statistically significant.

Statistical analyses were performed with STATA software version 12 (STATA Corp) and the R package *metafor*.

## CONFLICT OF INTEREST

AL and VDL have equity interest in a company producing medical food (L‐Nutra). This is not directly but indirectly related to the topic of this meta‐analysis. All other authors declare no competing interests.

## AUTHOR CONTRIBUTIONS

JR, AM, V Lagani, MB, and HZ carried out all statistical analysis. VDL and GP conceived the study. JR, AM, V Lagani, and FP participated in its design and coordination. The Ms was initially drafted by JR, AM, and VDL and then finalized by all authors. All the authors read and approved the final manuscript.

## Supporting information

Supplementary Material

## Data Availability

Extracted data for all included studies are available online. The analysis code is available. All figures and statistical outputs are available online.

## References

[acel13540-bib-0001] Andreassen, M. , Raymond, I. , Kistorp, C. , Hildebrandt, P. , Faber, J. , & Kristensen, L. Ø. (2009). IGF1 as predictor of all cause mortality and cardiovascular disease in an elderly population. European Journal of Endocrinology, 160(1), 25–31. 10.1530/EJE-08-0452 18931092

[acel13540-bib-0002] Bartke, A. , Sun, L. Y. , & Longo, V. (2013). Somatotropic signaling: Trade‐offs between growth, reproductive development, and longevity. Physiological Reviews, 93(2), 571–598. 10.1152/physrev.00006.2012 23589828 PMC3768106

[acel13540-bib-0003] Brioche, T. , Kireev, R. A. , Cuesta, S. , Gratas‐Delamarche, A. , Tresguerres, J. A. , Gomez‐Cabrera, M. C. , & Vina, J. (2014). Growth hormone replacement therapy prevents sarcopenia by a dual mechanism: Improvement of protein balance and of antioxidant defenses. The Journals of Gerontology Series A: Biological Sciences and Medical Sciences, 69(10), 1186–1198. 10.1093/gerona/glt187 24300031

[acel13540-bib-0004] Brugts, M. P. , van den Beld, A. W. , Hofland, L. J. , van der Wansem, K. , van Koetsveld, P. M. , Frystyk, J. , Lamberts, S. W. J. , & Janssen, J. A. M. J. L. (2008). Low circulating insulin‐like growth factor I bioactivity in elderly men is associated with increased mortality. The Journal of Clinical Endocrinology & Metabolism, 93(7), 2515–2522. 10.1210/jc.2007-1633 18413430

[acel13540-bib-0005] Burgers, A. M. G. , Biermasz, N. R. , Schoones, J. W. , Pereira, A. M. , Renehan, A. G. , Zwahlen, M. , Egger, M. , & Dekkers, O. M. (2011). Meta‐analysis and dose‐response metaregression: Circulating insulin‐like growth factor I (IGF‐I) and mortality. The Journal of Clinical Endocrinology & Metabolism, 96(9), 2912–2920. 10.1210/jc.2011-1377 21795450

[acel13540-bib-0006] Cappola, A. R. , Xue, Q.‐L. , Ferrucci, L. , Guralnik, J. M. , Volpato, S. , & Fried, L. P. (2003). Insulin‐like growth factor I and interleukin‐6 contribute synergistically to disability and mortality in older women. The Journal of Clinical Endocrinology & Metabolism, 88(5), 2019–2025. 10.1210/jc.2002-021694 12727948

[acel13540-bib-0007] Colombo, B. D. S. , Ronsoni, M. F. , Soares e Silva, P. E. , Fayad, L. , Wildner, L. M. , Bazzo, M. L. , Dantas‐Correa, E. B. , Narciso‐Schiavon, J. L. , & Schiavon, L. L. (2017). Prognostic significance of insulin‐like growth factor‐I serum levels in acute decompensation of cirrhosis. Biomarkers, 22(2), 127–132. 10.1080/1354750X.2016.1252949 27775431

[acel13540-bib-0008] DeBoer, M. D. , Scharf, R. J. , Leite, A. M. , Férrer, A. , Havt, A. , Pinkerton, R. , Lima, A. A. , & Guerrant, R. L. (2017). Systemic inflammation, growth factors, and linear growth in the setting of infection and malnutrition. Nutrition, 33, 248–253. 10.1016/j.nut.2016.06.013 27712965 PMC5193489

[acel13540-bib-0009] Delafontaine, P. , Song, Y.‐H. , & Li, Y. (2004). Expression, regulation, and function of IGF‐1, IGF‐1R, and IGF‐1 binding proteins in blood vessels. Arteriosclerosis, Thrombosis, and Vascular Biology, 24(3), 435–444. 10.1161/01.ATV.0000105902.89459.09 14604834

[acel13540-bib-0010] Duggan, C. , Wang, C.‐Y. , Neuhouser, M. L. , Xiao, L. , Smith, A. W. , Reding, K. W. , Baumgartner, R. N. , Baumgartner, K. B. , Bernstein, L. , Ballard‐Barbash, R. , & McTiernan, A. (2013). Associations of insulin‐like growth factor and insulin‐like growth factor binding protein‐3 with mortality in women with breast cancer. International Journal of Cancer, 132(5), 1191–1200. 10.1002/ijc.27753 22847383 PMC3764990

[acel13540-bib-0011] Feskanich, D. (2000). Prospective study of fruit and vegetable consumption and risk of lung cancer among men and women. Journal of the National Cancer Institute, 92(22), 1812–1823. 10.1093/jnci/92.22.1812 11078758

[acel13540-bib-0012] Fontana, L. , Partridge, L. , & Longo, V. D. (2010). Extending healthy life span—From yeast to humans. Science, 328(5976), 321–326. 10.1126/science.1172539 20395504 PMC3607354

[acel13540-bib-0013] Friedrich, N. , Haring, R. , Nauck, M. , Lüdemann, J. , Rosskopf, D. , Spilcke‐Liss, E. , Felix, S. B. , Dörr, M. , Brabant, G. , Völzke, H. , & Wallaschofski, H. (2009). Mortality and serum insulin‐like growth factor (IGF)‐I and IGF binding protein 3 concentrations. The Journal of Clinical Endocrinology & Metabolism, 94(5), 1732–1739. 10.1210/jc.2008-2138 19223521

[acel13540-bib-0014] Fulgoni, V. L. (2008). Current protein intake in America: Analysis of the National Health and Nutrition Examination Survey, 2003–2004. The American Journal of Clinical Nutrition, 87(5), 1554S–1557S. 10.1093/ajcn/87.5.1554S 18469286

[acel13540-bib-0015] Garnero, P. , Sornay‐Rendu, E. , & Delmas, P. D. (2000). Low serum IGF‐1 and occurrence of osteoporotic fractures in postmenopausal women. The Lancet, 355(9207), 898–899. 10.1016/S0140-6736(99)05463-X 10752709

[acel13540-bib-0016] Giovannucci, E. , Pollak, M. , Liu, Y. , Platz, E. A. , Majeed, N. , Rimm, E. B. , & Willett, W. C. (2003). Nutritional predictors of insulin‐like growth factor I and their relationships to cancer in men. Cancer Epidemiology, Biomarkers & Prevention, 12(2), 84–89. 12582016

[acel13540-bib-0017] Guevara‐Aguirre, J. , Balasubramanian, P. , Guevara‐Aguirre, M. , Wei, M. , Madia, F. , Cheng, C.‐W. , & Longo, V. D. (2011). Growth Hormone Receptor Deficiency Is Associated with a Major Reduction in Pro‐Aging Signaling, Cancer, and Diabetes in Humans. Science Translational Medicine, 3(70). 10.1126/scitranslmed.3001845 PMC3357623 21325617

[acel13540-bib-0018] Higashi, Y. , Sukhanov, S. , Anwar, A. , Shai, S.‐Y. , & Delafontaine, P. (2010). IGF‐1, oxidative stress and atheroprotection. Trends in Endocrinology & Metabolism, 21(4), 245–254. 10.1016/j.tem.2009.12.005 20071192 PMC2848911

[acel13540-bib-0019] Hu, D. , Pawlikowska, L. , Kanaya, A. , Hsueh, W.‐C. , Colbert, L. , Newman, A. B. , Satterfield, S. , Rosen, C. , Cummings, S. R. , Harris, T. B. , Ziv, E. , & for the Health, Aging, and Body Composition Study . (2009). Serum insulin‐like growth factor‐1 binding proteins 1 and 2 and mortality in older adults: The Health, Aging, and Body Composition Study: SERUM IGF‐1 BINDING PROTEINS 1 AND 2 AND MORTALITY IN OLDER ADULTS. Journal of the American Geriatrics Society, 57(7), 1213–1218. 10.1111/j.1532-5415.2009.02318.x 19558480 PMC2771612

[acel13540-bib-0020] Jia, T. , Gama Axelsson, T. , Heimbürger, O. , Bárány, P. , Lindholm, B. , Stenvinkel, P. , & Qureshi, A. R. (2014). IGF‐1 and survival in ESRD. Clinical Journal of the American Society of Nephrology, 9(1), 120–127. 10.2215/CJN.02470213 24178975 PMC3878692

[acel13540-bib-0021] Jimenez, A. G. (2016). Physiological underpinnings in life‐history trade‐offs in man’s most popular selection experiment: The dog. Journal of Comparative Physiology B, 186(7), 813–827. 10.1007/s00360-016-1002-4 27222254

[acel13540-bib-0022] Juul, A. , Dalgaard, P. , Blum, W. F. , Bang, P. , Hall, K. , Michaelsen, K. F. , Müller, J. , & Skakkebaek, N. E. (1995). Serum levels of insulin‐like growth factor (IGF)‐binding protein‐3 (IGFBP‐3) in healthy infants, children, and adolescents: The relation to IGF‐I, IGF‐II, IGFBP‐1, IGFBP‐2, age, sex, body mass index, and pubertal maturation. The Journal of Clinical Endocrinology & Metabolism, 80(8), 2534–2542. 10.1210/jcem.80.8.7543116 7543116

[acel13540-bib-0023] Katsanos, K. H. , Tsatsoulis, A. , Christodoulou, D. , Challa, A. , Katsaraki, A. , & Tsianos, E. V. (2001). Reduced serum insulin‐like growth factor‐1 (IGF‐1) and IGF‐binding protein‐3 levels in adults with inflammatory bowel disease. Growth Hormone & IGF Research, 11(6), 364–367. 10.1054/ghir.2001.0248 11914023

[acel13540-bib-0024] Kenyon, C. (2010). A pathway that links reproductive status to lifespan in *Caenorhabditis elegans*: Kenyon. Annals of the New York Academy of Sciences, 1204(1), 156–162. 10.1111/j.1749-6632.2010.05640.x 20738286

[acel13540-bib-0025] Key, T. J. (2011). Diet, insulin‐like growth factor‐1 and cancer risk. Proceedings of the Nutrition Society, 70(3), 385–388. 10.1017/S0029665111000127 21557887

[acel13540-bib-0026] Lenk, K. , Schuler, G. , & Adams, V. (2010). Skeletal muscle wasting in cachexia and sarcopenia: Molecular pathophysiology and impact of exercise training. Journal of Cachexia, Sarcopenia and Muscle, 1(1), 9–21. 10.1007/s13539-010-0007-1 PMC3060644 21475693

[acel13540-bib-0027] Levine, M. E. , Suarez, J. A. , Brandhorst, S. , Balasubramanian, P. , Cheng, C.‐W. , Madia, F. , Fontana, L. , Mirisola, M. G. , Guevara‐Aguirre, J. , Wan, J. , Passarino, G. , Kennedy, B. K. , Wei, M. , Cohen, P. , Crimmins, E. M. , & Longo, V. D. (2014). Low protein intake is associated with a major reduction in IGF‐1, cancer, and overall mortality in the 65 and younger but not older population. Cell Metabolism, 19(3), 407–417. 10.1016/j.cmet.2014.02.006 24606898 PMC3988204

[acel13540-bib-0028] Ma, J. , Pollak, M. N. , Giovannucci, E. , Chan, J. M. , Tao, Y. , Hennekens, C. H. , & Stampfer, M. J. (1999). Prospective study of colorectal cancer risk in men and plasma levels of insulin‐like growth factor (IGF)‐I and IGF‐binding protein‐3. JNCI Journal of the National Cancer Institute, 91(7), 620–625. 10.1093/jnci/91.7.620 10203281

[acel13540-bib-0029] Madsen, K. P. , Kjær, T. , Skinner, T. , & Willaing, I. (2019). Time preferences, diabetes self‐management behaviours and outcomes: A systematic review. Diabetic Medicine, 36(11), 1336–1348. 10.1111/dme.14102 31392757

[acel13540-bib-0030] Mao, K. , Quipildor, G. F. , Tabrizian, T. , Novaj, A. , Guan, F. , Walters, R. O. , Delahaye, F. , Hubbard, G. B. , Ikeno, Y. , Ejima, K. , Li, P. , Allison, D. B. , Salimi‐Moosavi, H. , Beltran, P. J. , Cohen, P. , Barzilai, N. , & Huffman, D. M. (2018). Late‐life targeting of the IGF‐1 receptor improves healthspan and lifespan in female mice. Nature Communications, 9(1), 2394. 10.1038/s41467-018-04805-5 PMC6008442 29921922

[acel13540-bib-0031] Miyake, H. , Kanazawa, I. , & Sugimoto, T. (2016). Decreased serum insulin‐like growth factor‐I level is associated with the increased mortality in type 2 diabetes mellitus. Endocrine Journal, 63(9), 811–818. 10.1507/endocrj.EJ16-0076 27349183

[acel13540-bib-0032] Nashiro, K. , Guevara‐Aguirre, J. , Braskie, M. N. , Hafzalla, G. W. , Velasco, R. , Balasubramanian, P. , & Longo, V. D. (2017). Brain Structure and Function Associated with Younger Adults in Growth Hormone Receptor‐Deficient Humans. The Journal of Neuroscience, 37(7), 1696–1707. 10.1523/JNEUROSCI.1929-16.2016 28073935 PMC5320603

[acel13540-bib-0033] Orsini, N. (2010). From floated to conventional confidence intervals for the relative risks based on published dose–response data. Computer Methods and Programs in Biomedicine, 98(1), 90–93. 10.1016/j.cmpb.2009.11.005 19959250

[acel13540-bib-0034] Plan and operation of the Third National Health and Nutrition Examination Survey. 1988‐94. Series 1: Programs and collection procedures. (1994). Vital and Health Statistics. Ser. 1, Programs and Collection Procedures, (32), 1–407. 7975354

[acel13540-bib-0035] Podshivalova, K. , Kerr, R. A. , & Kenyon, C. (2017). How a mutation that slows aging can also disproportionately extend end‐of‐life decrepitude. Cell Reports, 19(3), 441–450. 10.1016/j.celrep.2017.03.062 28423308 PMC5526670

[acel13540-bib-0036] Renehan, A. G. , Zwahlen, M. , Minder, C. , O’Dwyer, S. T. , Shalet, S. M. , & Egger, M. (2004). Insulin‐like growth factor (IGF)‐I, IGF binding protein‐3, and cancer risk: Systematic review and meta‐regression analysis. The Lancet, 363(9418), 1346–1353. 10.1016/S0140-6736(04)16044-3 15110491

[acel13540-bib-0037] Saki, N. , Abroun, S. , Salari, F. , Rahim, F. , Shahjahani, M. , & Mohammadi‐Asl, J. (2017). Molecular Aspects of Bone Resorption in β‐Thalassemia Major. Cell Journal (Yakhteh), 17(2), 10.22074/cellj.2016.3713 PMC4503833 26199898

[acel13540-bib-0038] Shi, R. , Berkel, H. J. , & Yu, H. (2001). Insulin‐like growth factor‐I and prostate cancer: A meta‐analysis. British Journal of Cancer, 85(7), 991–996. 10.1054/bjoc.2001.1961 11592771 PMC2375097

[acel13540-bib-0039] Stang, A. (2010). Critical evaluation of the Newcastle‐Ottawa scale for the assessment of the quality of nonrandomized studies in meta‐analyses. European Journal of Epidemiology, 25(9), 603–605. 10.1007/s10654-010-9491-z 20652370

[acel13540-bib-0040] Steuerman, R. , Shevah, O. , & Laron, Z. (2011). Congenital IGF1 deficiency tends to confer protection against post‐natal development of malignancies. European Journal of Endocrinology, 164(4), 485–489. 10.1530/EJE-10-0859 21292919

[acel13540-bib-0041] Street, M. E. , de’Angelis, G. L. , Camacho‐Hübner, C. , Giovannelli, G. , Ziveri, M. A. , Bacchini, P. L. , Bernasconi, S. , Sansebastiano, G. , & Savage, M. O. (2004). Relationships between serum IGF‐1, IGFBP‐2, interleukin‐1Beta and Interleukin‐6 in inflammatory bowel disease. Hormone Research in Paediatrics, 61(4), 159–164. 10.1159/000075699 14691340

[acel13540-bib-0042] Street, M. E. , Ziveri, M. A. , Spaggiari, C. , Viani, I. , Volta, C. , Grzincich, G. L. , Virdis, R. , & Bernasconi, S. (2006). Inflammation is a modulator of the insulin‐like growth factor (IGF)/IGF‐binding protein system inducing reduced bioactivity of IGFs in cystic fibrosis. European Journal of Endocrinology, 154(1), 47–52. 10.1530/eje.1.02064 16381990

[acel13540-bib-0043] Sun, L. Y. , Fang, Y. , Patki, A. , Koopman, J. J. E. , Allison, D. B. , Hill, C. M. , Masternak, M. M. , Darcy, J. , Wang, J. , McFadden, S. , & Bartke, A. (2017). Longevity is impacted by growth hormone action during early postnatal period. eLife, 6, e24059. 10.7554/eLife.24059 28675141 PMC5515575

[acel13540-bib-0044] United States Department of Health and Human Services. Centers For Disease Control And Prevention. National Center For Health Statistics (1998). National Health and Nutrition Examination Survey III, 1988–1994: Version 1. [Data set]. ICPSR ‐ Interuniversity Consortium for Political and Social Research, 10.3886/ICPSR02231.V1

[acel13540-bib-0045] Vinciguerra, M. , Santini, M. P. , Claycomb, W. C. , Ladurner, A. G. , & Rosenthal, N. (2009). Local IGF‐1 isoform protects cardiomyocytes from hypertrophic and oxidative stresses via SirT1 activity. Aging, 2(1), 43–62. 10.18632/aging.100107 20228935 PMC2837204

[acel13540-bib-0046] Vinciguerra, M. , Santini, M. P. , Martinez, C. , Pazienza, V. , Claycomb, W. C. , Giuliani, A. , & Rosenthal, N. (2012). mIGF‐1/JNK1/SirT1 signaling confers protection against oxidative stress in the heart: MIGF‐1/SirT1 signaling and cardiac oxidative stress. Aging Cell, 11(1), 139–149. 10.1111/j.1474-9726.2011.00766.x 22051242

[acel13540-bib-0047] Watling, C. Z. , Kelly, R. K. , Tong, T. Y. N. , Piernas, C. , Watts, E. L. , Tin Tin, S. , Knuppel, A. , Schmidt, J. A. , Travis, R. C. , Key, T. J. , & Perez‐Cornago, A. (2021). Associations of circulating insulin‐like growth factor‐I with intake of dietary proteins and other macronutrients. Clinical Nutrition, 40(7), 4685–4693. 10.1016/j.clnu.2021.04.021 34237695 PMC8345002

[acel13540-bib-0048] Willett, W. C. , & Ludwig, D. S. (2020). Milk and health. New England Journal of Medicine, 382(7), 644–654. 10.1056/NEJMra1903547 32053300

[acel13540-bib-0049] Yamamoto, M. , Iguchi, G. , Fukuoka, H. , Suda, K. , Bando, H. , Takahashi, M. , Nishizawa, H. , Seino, S. , & Takahashi, Y. (2013). SIRT1 regulates adaptive response of the growth hormone—Insulin‐like growth factor‐I axis under fasting conditions in liver. Proceedings of the National Academy of Sciences of the United States of America, 110(37), 14948–14953. 10.1073/pnas.1220606110 23980167 PMC3773795

[acel13540-bib-0050] Yu, H. , Spitz, M. R. , Mistry, J. , Gu, J. , Hong, W. K. , & Wu, X. (1999). Plasma levels of insulin‐like growth factor‐I and lung cancer risk: A case‐control analysis. JNCI Journal of the National Cancer Institute, 91(2), 151–156. 10.1093/jnci/91.2.151 9923856

