# Supplementary material for: Association between IGF‐1 levels ranges and all‐cause mortality: A meta‐analysis
Source: Aging Cell. 2022 Jan 20;21(2):e13540. doi: 10.1111/acel.13540 (PMC8844108; doi:10.1111/acel.13540)
Supplement: Supplementary file 1 — Supplementary Material [file ACEL-21-e13540-s001.pdf]

Supporting information

Association Between IGF-1 Levels Ranges and All-cause Mortality: A Meta-Analysis

Jamal Rahmani[a$], Alberto Montesanto[b$], Edward Giovannucci[c], Hamid Zand[d], Meisam Barati[d], John J. Kopchick[e], Mario G. Mirisola[f], Vincenzo Lagani[g,h], Hiba Bawadi[i], Raffaele Vardavas[j], Alessandro Laviano[k], Kaare Christensen[l], Giuseppe Passarino#[2], Valter D. Longo#[m,n]*

Table of Contents of supporting information

Supplementary methods 2

Supplementary figures 3

Supplementary tables 5

MOOSE checklist 8

Supplementary references 11

Supplementary methods

Sensitivity analysis of the adopted IGF-1 interval

To investigate the sensitivity of our results with respect to the chosen optimal IGF-1 interval (120-160 ng/ml), we repeated the meta-analysis by adopting a larger interval, 100-180. The enlargement led to the inclusion of two more studies, namely Rowlands and colleagues and Kaplan and colleagues (Kaplan et al., 2017; Rowlands et al., 2012). Figure S1 reports the results of this analysis: the combined hazard ratios for both contrasts (A: low versus middle; B: high versus middle IGF-1 categories) remain statistically significant, and their absolute values were identical or very close to the results obtained with the 120-160 IGF-1 optimal interval (Figure 3 D-E).

Supplementary figures

Figure S1: Forest plot of pooled analysis for the low (A) and the high (B) categories of IGF-1 compared to the middle category (IGF-1 interval 100-180 ng/ml).

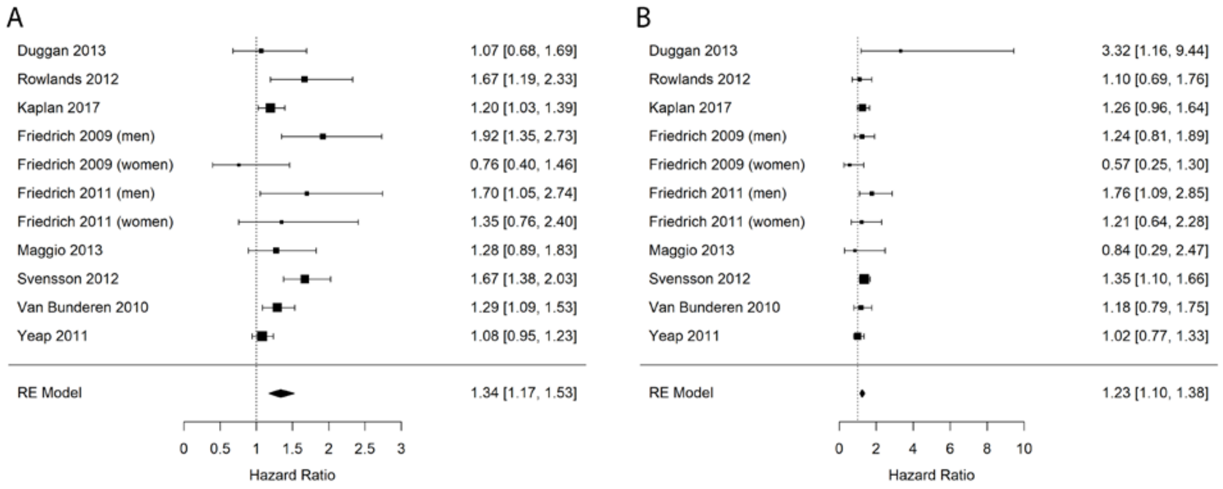

Figure S2: Funnel plot of all studies with a pseudo 95% confidence interval.

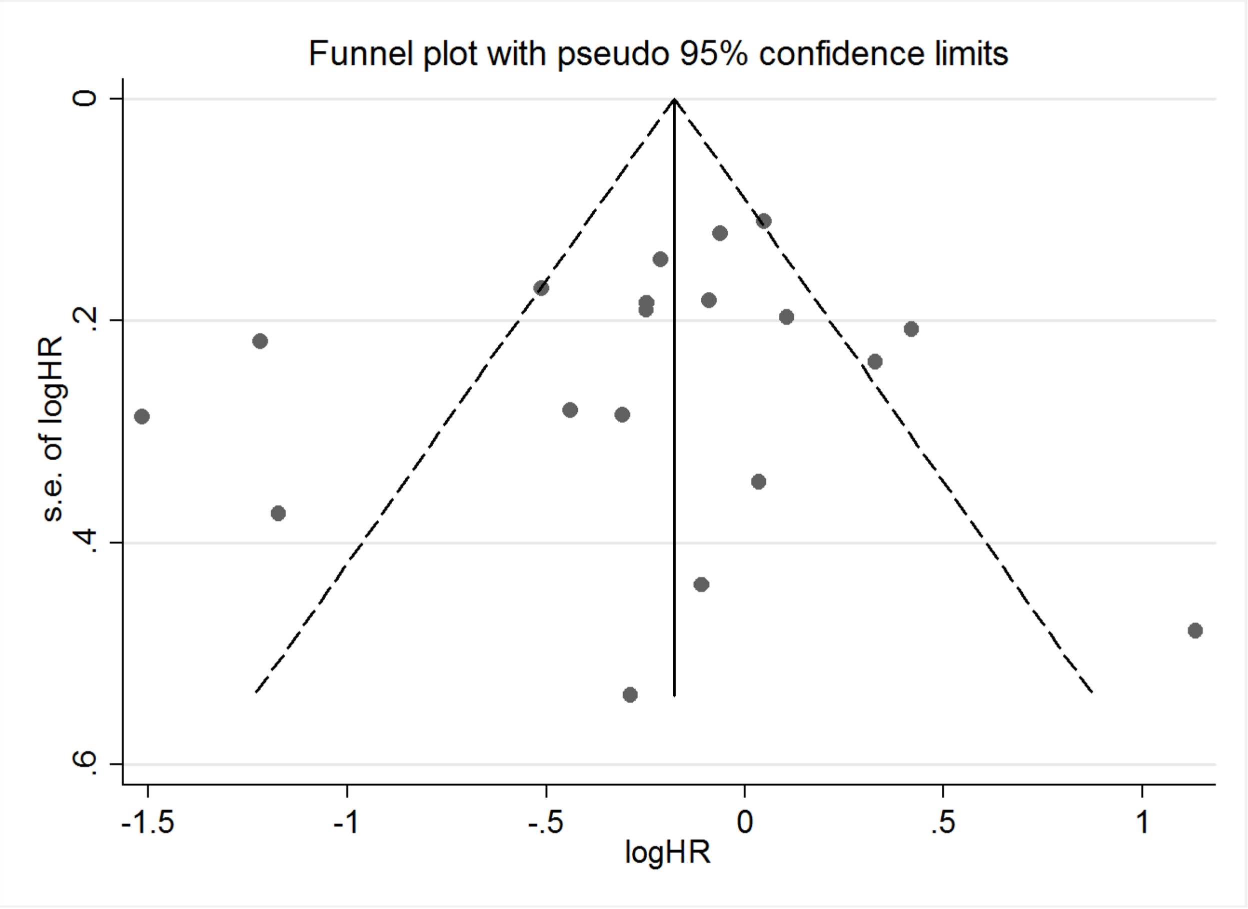

Figure S3: Sensitivity analysis

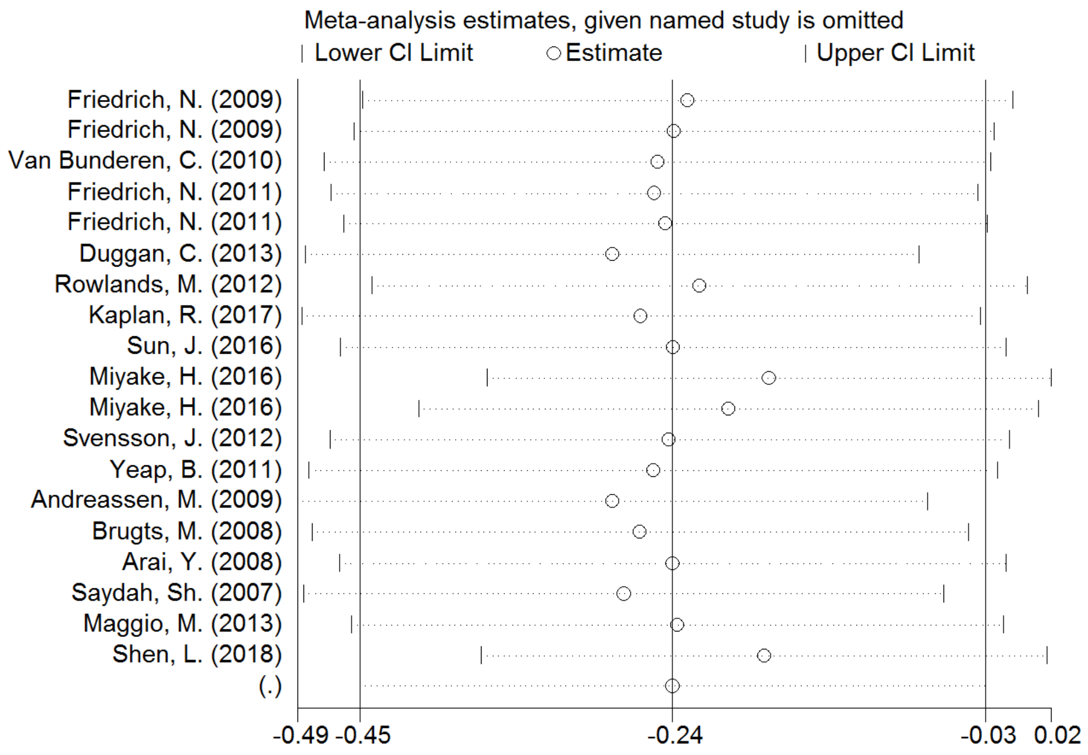

Supplementary Tables

Table S1: Used words in literature search

PubMed/MEDLINE	Cochrane library	Scopus
(((((("Insulin-Like Growth Factor I"[Mesh]) OR Insulin-Like Growth Factor I[Title/Abstract]) OR IGF-1[Title/Abstract]) OR IGF1[Title/Abstract])) AND (((("Mortality"[Mesh]) OR (mortality[tiab]) OR ("Death"[Mesh]) OR ("Death"[tiab]) OR ("Deaths"[tiab]) OR ("fatal"[tiab]) OR ("Survival"[Mesh]) OR ("Survival"[tiab]))))) AND ((((prospective[Title/Abstract]) OR longitudinal[Title/Abstract]) OR follow-up[Title/Abstract]) OR cohort[Title/Abstract])	(Insulin-Like Growth Factor I OR IGF-1 OR IGF1) AND (mortality OR Death OR Fatal OR Survival) AND (prospective OR longitudinal OR follow-up OR Cohort)	((TITLE-ABS-KEY (insulin like AND growth AND factor AND 1) OR TITLE-ABS-KEY (IGF-1) OR TITLE-ABS-KEY (IGF1))) AND ((TITLE-ABS-KEY (mortality) OR TITLE-ABS-KEY (death) OR TITLE-ABS-KEY (deaths) OR TITLE-ABS-KEY (fatal) OR TITLE-ABS-KEY (survival))) AND ((TITLE-ABS-KEY (prospective) OR TITLE-ABS-KEY (longitudinal) OR TITLE-ABS-KEY (follow-up) OR TITLE-ABS-KEY (cohort)))

Table S2: The nineteen selected studies with the covariates used by each one.

Study	Covariates
(Nele Friedrich et al., 2009)	Sex, WC and physical activity. age was used as time scale.
(Nele Friedrich et al., 2009)	Sex, WC and physical activity. age was used as time scale.
(van Bunderen et al., 2010)	sex, age, BMI, smoking, alcohol consumption, DM, physical activity, and albumin
(N. Friedrich et al., 2011)	Sex, BMI, smoking, physical activity, liver disease, and ischemic heart disease. age was used as time scale.
(N. Friedrich et al., 2011)	Sex, BMI, smoking, physical activity, liver disease. age was used as time scale.
(Duggan et al., 2013)	Sex, BMI, race; tamoxifen use , Treatment received
(Rowlands et al., 2012)	Sex,age, treatment status, smoking, PSA, Gleason grade and mutual IGF
(Kaplan et al., 2017)	age, sex, race, smoking, alcohol consumption, status of general health, hypertension, prehypertension, physical activity, protein consumption as caloric intake, estrogen and progestin hormone consumption, serum albumin concentration, serum cystatin concentration, hs-CRP, BMI
(Sun et al., 2016)	age, sex, DM, smoking, PEW, and eGFR
(Miyake, Kanazawa, & Sugimoto, 2016)	Sex, age, duration of diabetes, BMI, HbA1c, and serum creatinine, serum albumin, systolic blood pressure, ALT, LDL-cholesterol, smoking, and past history of CVD
(Miyake et al., 2016)	Sex, age, duration of diabetes, BMI, HbA1c, and serum creatinine, serum albumin, systolic blood pressure, ALT, LDL-cholesterol and smoking

(Svensson et al., 2012)	Sex, age, MrOS site, and time of serum sampling
(Yeap et al., 2011)	Sex, age, BMI, waist:hip ratio, smoking, fasting status, dyslipidemia, hypertension and medical comorbidities
(Andreassen et al., 2009)	age, sex, history of hypertension, DM, atrial fibrillation, smoking, total cholesterol and log NT-proBNP, history of ischemic heart disease (IHD), stroke, TIA or CHF
(Brugts et al., 2008)	Sex, age, BMI, smoking, SBP, diabetes, LDL, and HDL
(Arai et al., 2008)	age, sex, education, smoking, Barthel Index, CDR scale, numbers of comorbidities, and serum levels of albumin, HDL-C, and IL-6
(Saydah, Graubard, Ballard-Barbash, & Berrigan, 2007)	age, sex, race, smoking, alcohol, MI, and insulin-like growth factor binding protein
(Maggio, 2007)	age, sex, caloric intake, GOT, fasting insulin, body mass index, dehydroepiandrosterone sulfate, testosterone, interleukin-6, congestive heart failure, stroke, physical activity, and IGFBP-1.
(Shen, Xu, Zhang, & Jiang, 2018)	age, sex, HBsAg-positive status, HCV Ab-positive status, first-degree history of LCC, number of tumors, tumor size, present cirrhosis, vascular invasion, distant metastasis, presence of PVT, lymph node involvement, Child–Pugh class, TNM stage, blood levels of ALT, AST, total bilirubin, AFP, Hs-CRP, and IGF-1.

Table S3. Quality assessment of the studies included in this meta-analysis.

Quality assessment criteria		Included studies																		
		[(3)] (women)	[(3)] (men)	[(4)]	[(5)] (women)	[(5)] (men)	[(6)]	[(2)]	[(1)]	[(7)]	[(8)]	[(8)]	[(9)]	[(4)]	[(11)]	[(12)]	[(13)]	[(14)]	[(15)]	[(16)]
Selection	Representativeness of exposed cohort?	1	1	1	1	1	1	1	1	1	1	1	1	1	1	1	1	1	1	1
	Selection of the non-exposed cohort?	1	1	1	1	1	1	1	1	1	1	1	1	1	1	1	1	1	1	1
	Ascertainment of exposure?	1	1	1	1	1	1	1	1	1	1	1	1	1	1	1	1	1	1	1
	Demonstration that outcome of interest was not present at start of study?	1	1	1	1	1	1	1	1	1	1	1	1	1	1	1	1	1	1	1
Comparability	Study controls for age/sex?	0	0	1	0	0	0	0	1	1	1	1	0	1	1	1	1	1	0	1
	Study controls for at least 3 additional risk factors?	0	0	1	1	1	0	0	1	1	1	1	0	1	1	1	1	1	0	0
Outcome	Assessment of outcome?	1	1	1	1	1	1	1	1	1	1	1	1	1	1	1	1	1	1	1
	Was follow-up long enough for outcome to occur?	1	1	1	1	1	1	0	1	0	1	1	1	1	1	1	1	1	1	0
	Adequacy of follow-up of cohorts?	1	1	1	1	1	1	1	1	1	1	1	1	1	1	1	1	1	1	1
**Overall Quality Score (Maximum = 9)**		7	7	9	8	8	7	6	9	8	9	9	7	9	9	9	9	9	7	7

MOOSE checklist

Meta-analysis of Observational Studies in Epidemiology.

	Reported on page	Comments
**Reporting of background should include**		
Problem definition	5	
Hypothesis statement	6	
Description of study outcomes	6	
Type of exposure or intervention used	6	
Type of study designs used	7	
Study population	6	
**Reporting of search strategy should include**		
Qualifications of searchers (eg librarians and investigators)	6	
Search strategy, including time period used in the synthesis and key words	6	
Effort to include all available studies, including contact with authors	6	
Databases and registries searched	6	
Search software used, name and version, including special features used (eg explosion)	8	
Use of hand searching (eg reference lists of obtained articles)	7	
List of citations located and those excluded, including justification	7	
Method of addressing articles published in languages other than English	6	
Method of handling abstracts and unpublished studies	6	
Description of any contact with authors	6	
**Reporting of methods should include**		
Description of relevance or appropriateness of studies assembled for assessing the hypothesis to be tested	8	
Rationale for the selection and coding of data (eg sound clinical principles or convenience)	8	
Documentation of how data were classified and coded (eg multiple raters, blinding and interrater reliability)	8	
Assessment of confounding (eg comparability of cases and controls in studies where appropriate)	8	
Assessment of study quality, including blinding of quality assessors, stratification or regression on possible predictors of study results	7	
Assessment of heterogeneity	9	

Description of statistical methods (eg complete description of fixed or random effects models, justification of whether the chosen models account for predictors of study results, dose-response models, or cumulative meta-analysis) in sufficient detail to be replicated	9	
Provision of appropriate tables and graphics	9	
**Reporting of results should include**		
Graphic summarizing individual study estimates and overall estimate	Figure2	
Table giving descriptive information for each study included	Table 1	
Results of sensitivity testing (eg subgroup analysis)	10	
Indication of statistical uncertainty of findings	11	
**Reporting of discussion should include**		
Quantitative assessment of bias (eg publication bias)	10	
Justification for exclusion (eg exclusion of non-English language citations)	6	
Assessment of quality of included studies	9	
**Reporting of conclusions should include**		
Consideration of alternative explanations for observed results	12	
Generalization of the conclusions (eg appropriate for the data presented and within the domain of the literature review)	12-15	
Guidelines for future research	15	
Disclosure of funding source	16	

Supplementary references

Andreassen, M., Raymond, I., Kistorp, C., Hildebrandt, P., Faber, J., & Kristensen, L. Ø. (2009). IGF1 as predictor of all cause mortality and cardiovascular disease in an elderly population. *European Journal of Endocrinology*, *160*(1), 25–31. doi: 10.1530/EJE-08-0452

Arai, Y., Takayama, M., Gondo, Y., Inagaki, H., Yamamura, K., Nakazawa, S., … Hirose, N. (2008). Adipose Endocrine Function, Insulin-Like Growth Factor-1 Axis, and Exceptional Survival Beyond 100 Years of Age. *The Journals of Gerontology Series A: Biological Sciences and Medical Sciences*, *63*(11), 1209–1218. doi: 10.1093/gerona/63.11.1209

Brugts, M. P., van den Beld, A. W., Hofland, L. J., van der Wansem, K., van Koetsveld, P. M., Frystyk, J., … Janssen, J. A. M. J. L. (2008). Low Circulating Insulin-Like Growth Factor I Bioactivity in Elderly Men Is Associated with Increased Mortality. *The Journal of Clinical Endocrinology & Metabolism*, *93*(7), 2515–2522. doi: 10.1210/jc.2007-1633

Duggan, C., Wang, C.-Y., Neuhouser, M. L., Xiao, L., Smith, A. W., Reding, K. W., … McTiernan, A. (2013). Associations of insulin-like growth factor and insulin-like growth factor binding protein-3 with mortality in women with breast cancer. *International Journal of Cancer*, *132*(5), 1191–1200. doi: 10.1002/ijc.27753

Friedrich, N., Schneider, H., Dörr, M., Nauck, M., Völzke, H., Klotsche, J., … Stalla, G. K. (2011). All-cause mortality and serum insulin-like growth factor I in primary care patients. *Growth Hormone & IGF Research*, *21*(2), 102–106. doi: 10.1016/j.ghir.2011.02.003

Friedrich, Nele, Haring, R., Nauck, M., Lüdemann, J., Rosskopf, D., Spilcke-Liss, E., … Wallaschofski, H. (2009). Mortality and Serum Insulin-Like Growth Factor (IGF)-I and IGF Binding Protein 3 Concentrations. *The Journal of Clinical Endocrinology & Metabolism*, *94*(5), 1732–1739. doi: 10.1210/jc.2008-2138

Kaplan, R. C., Strizich, G., Aneke-Nash, C., Dominguez-Islas, C., Bůžková, P., Strickler, H., … Newman, A. (2017). Insulinlike Growth Factor Binding Protein-1 and Ghrelin Predict Health Outcomes Among Older Adults: Cardiovascular Health Study Cohort. *The Journal of Clinical Endocrinology & Metabolism*, *102*(1), 267–278. doi: 10.1210/jc.2016-2779

Maggio, M. (2007). Relationship Between Low Levels of Anabolic Hormones and 6-Year Mortality in Older MenThe Aging in the Chianti Area (InCHIANTI) Study. *Archives of Internal Medicine*, *167*(20), 2249. doi: 10.1001/archinte.167.20.2249

Miyake, H., Kanazawa, I., & Sugimoto, T. (2016). Decreased serum insulin-like growth factor-I level is associated with the increased mortality in type 2 diabetes mellitus. *Endocrine Journal*, *63*(9), 811–818. doi: 10.1507/endocrj.EJ16-0076

Rowlands, M.-A., Holly, J. M. P., Hamdy, F., Phillips, J., Goodwin, L., Marsden, G., … Martin, R. M. (2012). Serum insulin-like growth factors and mortality in localised and advanced clinically detected prostate cancer. *Cancer Causes & Control*, *23*(2), 347–354. doi: 10.1007/s10552-011-9883-8

Saydah, S., Graubard, B., Ballard-Barbash, R., & Berrigan, D. (2007). Insulin-like Growth Factors and Subsequent Risk of Mortality in the United States. *American Journal of Epidemiology*, *166*(5), 518–526. doi: 10.1093/aje/kwm124

Shen, L., Xu, L., Zhang, J., & Jiang, D. (2018). Preoperative Serum Insulin-Like Growth Factor 1 Level as a Prognostic Factor in Patients Undergoing Hepatic Resection for Hepatocellular Carcinoma. *Journal of Interferon & Cytokine Research*, *38*(4), 153–160. doi: 10.1089/jir.2017.0107

Sun, J., Axelsson, J., Machowska, A., Heimbürger, O., Bárány, P., Lindholm, B., … Qureshi, A. R. (2016). Biomarkers of Cardiovascular Disease and Mortality Risk in Patients with Advanced CKD. *Clinical Journal of the American Society of Nephrology*, *11*(7), 1163–1172. doi: 10.2215/CJN.10441015

Svensson, J., Carlzon, D., Petzold, M., Karlsson, M. K., Ljunggren, Ö., Tivesten, Å., … Ohlsson, C. (2012). Both Low and High Serum IGF-I Levels Associate with Cancer Mortality in Older Men. *The Journal of Clinical Endocrinology & Metabolism*, *97*(12), 4623–4630. doi: 10.1210/jc.2012-2329

van Bunderen, C. C., van Nieuwpoort, I. C., van Schoor, N. M., Deeg, D. J. H., Lips, P., & Drent, M. L. (2010). The Association of Serum Insulin-Like Growth Factor-I with Mortality, Cardiovascular Disease, and Cancer in the Elderly: A Population-Based Study. *The Journal of Clinical Endocrinology & Metabolism*, *95*(10), 4616–4624. doi: 10.1210/jc.2010-0940

Yeap, B. B., Chubb, S. A. P., McCaul, K. A., Ho, K. K. Y., Hankey, G. J., Norman, P. E., & Flicker, L. (2011). Associations of IGF1 and IGFBPs 1 and 3 with all-cause and cardiovascular mortality in older men: The Health In Men Study. *European Journal of Endocrinology*, *164*(5), 715–723. doi: 10.1530/EJE-11-0059
